# Will an Unsupervised Self-Testing Strategy Be Feasible to Operationalize in Canada? Results from a Pilot Study in Students of a Large Canadian University

**DOI:** 10.1155/2014/747619

**Published:** 2014-01-09

**Authors:** Nitika Pant Pai, Madhavi Bhargava, Lawrence Joseph, Jigyasa Sharma, Sabrina Pillay, Bhairavi Balram, Pierre-Paul Tellier

**Affiliations:** ^1^Department of Medicine, McGill University, Montreal, QC, Canada H3A 1A1; ^2^Division of Clinical Epidemiology, McGill University and Health Centre, Montreal, QC, Canada H3A 1A1; ^3^Department of Epidemiology, Biostatistics & Occupational Health, McGill University, Montreal, QC, Canada H3A 1A2; ^4^McGill University Student Health Services, Montreal, QC, Canada H3A 0G3

## Abstract

*Background.* A convenient, private, and accessible HIV self-testing strategy stands to complement facility-based conventional testing. Over-the-counter oral HIV self-tests are approved and available in the United States, but not yet in Canada. Canadian data on self-testing is nonexistent. We investigated the feasibility of offering an unsupervised self-testing strategy to Canadian students. *Methods.* Between September 2011 and May 2012, we recruited 145 students from a student health clinic of a large Canadian university. Feasibility of operationalization (i.e., self-test conduct, acceptability, convenience, and willingness to pay) was evaluated. Self-test conduct was computed with agreement between the self-test performed by the student and the test repeated by a healthcare professional. Other metrics were measured on a survey. * Results.* Participants were young (median age: 22 years), unmarried (97%), and 47% were out of province or international students. Approximately 52% self-reported a history of unprotected casual sex and sex with multiple partners. Self-test conduct agreement was high (100%), so were acceptability (81%), convenience (99%), and willingness to pay (74%) for self-tests. Concerns included accuracy of self-tests and availability of expedited linkages. *Conclusion.* An unsupervised self-testing strategy was found to be feasible in Canadian students. Findings call for studies in at-risk populations to inform Canadian policy.

## 1. Introduction

At the end of 2008, an estimated 65,000 Canadians were living with HIV, an increase of 14% since 2005 [1]. About 2,300 to 4,300 new HIV infections occurred in 2008 alone. Despite the availability of facility-based HIV testing and counselling [2], approximately 26% of all positive Canadians remain unaware of their HIV serostatus [1]. Systemic and social barriers, such as stigma, perceived discrimination, and the fear of social visibility and lack of confidentiality have long deterred uptake of conventional facility-based testing. In addition, fear of the test result and the belief that they are not at risk can also prevent some individuals from accessing testing. Furthermore, an increased anxiety associated with long wait times to receive test results and loss of work days associated with getting an HIV test have further impeded the uptake of facility-based HIV testing [3–9]. Conventional testing at a healthcare facility in Canada can be (a) nominal (i.e., the name of the person being tested appears on test forms, results, and medical records), (b) nonnominal (i.e., the test is ordered using a code, but the result is recorded in the patient's medical records), or (c) anonymous (i.e., a code is used instead of the name of the patient). Anonymous testing is offered in only seven Canadian provinces. Notification of HIV positivity is mandatory in all provinces.

With the recent approval by the Food and Drug Administration (FDA) of an over-the-counter (OTC) oral HIV self-test [10] in the United States (US), the time is ripe to address similar approvals of these tests in Canada. To date, only one point-of-care (POC) test for HIV (INSTI HIV-1 Antibody Test) has been approved in Canada [11], and it is blood-based—no oral HIV POC tests are approved for sale yet in Canada. However, offering HIV self-tests OTC in pharmacies for use at home has the potential to engage more people in self-testing. To date, data on acceptability of such tests remains limited. Offering a confidential and private self-testing option as in the US [4, 8] is a possibility in Canada, provided linked counselling and referral systems are in place to ensure linkage to care. The assumption is that the empowerment, decreased social visibility, privacy, and confidentiality offered by self-tests [12, 13] could increase the uptake of HIV testing and bring more individuals into care, although evidence to support or discredit this assumption remains limited. At the health systems level, however, in-home HIV self-testing could make notification and surveillance for HIV challenging and could potentially lead to an underestimation of HIV infection rates [14]. In addition, erroneous, invalid, indeterminate, or false reactive self-test results could occur. In the United States, it has been reported that some individuals failed to read their self-test results accurately [10]. The process of self-testing, as available in the US, assumes individuals are motivated to buy the test, possess sufficient literacy to accurately understand the instructions, conduct the test, and interpret their result. Furthermore, individuals need to be proactive enough to call a toll-free number for posttest counselling and linkages to staging, treatment, and care. If any of the steps is incorrectly done, then perhaps the goal of self-testing would not be achieved. In addition, the risk of adverse events cannot be ignored. These can range from coercive testing to extreme emotional response and suicide, although it has been argued that these issues are associated more with a diagnosis of HIV and could therefore occur even with conventional testing [13]. Global data that refute or validate negative concerns with self-testing are currently limited, and data on self-test conduct, preference, and willingness to pay by prospective self-testers are nonexistent in the Canadian context.

In a recent systematic review, we documented two self-testing strategies evaluated globally: supervised and unsupervised [15–22]. In a supervised self-testing strategy, testing and counselling processes are aided at all times by a healthcare professional (HCP). In an unsupervised self-testing strategy, the self-tester performs the self-test on his own without any help, and counselling and linkages are offered either online or over the phone by trained counsellors or through pharmacies. In this study, we assessed the feasibility of operationalization of an unsupervised self-testing strategy in a population of students attending a large Canadian university in Montreal.

## 2. Methods 

Feasibility of operationalization was defined by the following end points: (a) agreement of self-test conduct and (b) acceptability. Our primary end point was to observe the feasibility of self-test conduct, defined by the agreement between the oral self-test performed and interpreted by the student versus the oral test performed and interpreted by the HCP as a POC test on-site.

Self-test conduct and performance included self-test sample collection (swabbing), placement of the test device into the developer solution, self-interpretation of the test result, and recording of test results. This recorded result was compared with the repeat test performed subsequently by the HCP on the student on-site.

Our secondary end point of acceptability was documented by a questionnaire based survey that enquired about a student's acceptability, preferences, willingness to pay, concerns, and challenges with the use of self-tests in the Canadian context.

For the period of September 2011 to May 2012, we conducted a cross-sectional study at the student health services clinic of a large Canadian university. The clinic is known for its past successes with HIV rapid testing initiatives and has trained nurses and counsellors on-site who provide rapid POC testing for HIV. At the time when this study was conducted, the OTC test was not approved by the FDA. Since the test is not yet approved by health authorities in Canada, it was considered an “investigational device for research use only,” which meant that it could not be used for clinical decision-making. Hence, the test was used at the clinic (an approved health facility), and the study had to be conducted only with participants that were confirmed seronegatives (because of the investigational nature of the device, the test could not be used with participants of unknown serostatus). Because of this limitation, we were unable to identify new infections using the self-test, and so estimates of accuracy could not be computed.

Eligibility criteria included students (both males and females), aged 18 years or older, willing to provide informed consent in English or French. All recruited participants had to undergo a HIV laboratory test as a first step, and once results were communicated to participants, only confirmed HIV negative students were invited to participate in the next steps of the study. The study was approved by the Institutional Review Board of the McGill University Health Centre, Montreal.

**Figure 1:**
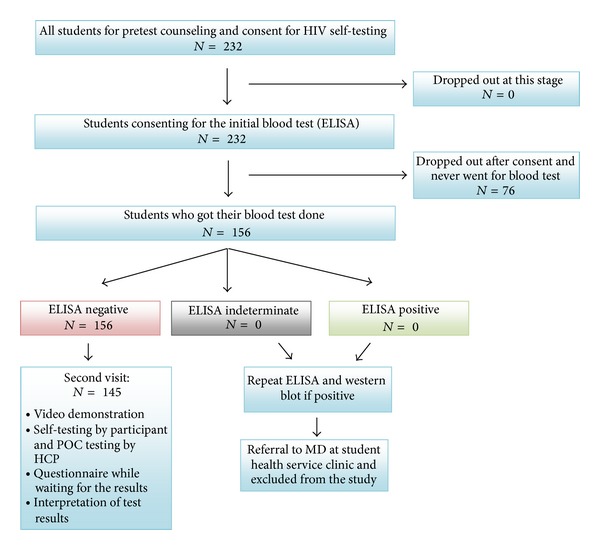
Flow of participants and test algorithm.

Figure 1 explains the flow of participants in the study. Each participant visited the clinic twice during the study. In the first visit, pretest counselling was offered and informed consent was obtained. Participants were provided with details on study procedures and their blood was drawn for conventional HIV testing to confirm their serostatus before inviting them to participate in the next steps of the study. Instructions on using the self-test (OraQuick Rapid HIV-1/2 Antibody Test, OraSure Technologies, Inc., PA, USA) were also provided.

The clinic staff contacted confirmed seronegative study participants to schedule their second visit after receiving their HIV result; participants found to be seropositive were contacted as soon as their confirmatory results were available to be linked to care. In the second visit, prior to testing, the students were provided with pretest questionnaires (QES), a self-test kit, a pamphlet that contained information on HIV and risk reduction, a timer, and a pictorial reference guide on self-testing that outlined all the steps for self-testing. Test instructions were also available as a video, which could be played with one of the portable DVD players provided on-site.

All oral self-tests were performed in the privacy of an examination room located inside the clinic. The self-testing procedure consisted of performing the self-test and then interpreting and recording its result after 20 minutes. The HCP was not present during the self-test but was available if needed. After completion of the self-testing procedure, participants sought the HCP, who then performed the test on the student, then interpreted and recorded the result in the student's presence. The self-test session was followed by a posttest risk reduction counselling session led by the HCP. Students were then asked to complete a posttest questionnaire on their experience with self-testing.

The bilingual (English and French) pre- and posttest QES that was filled in by students themselves collected information on demographics (e.g., age, gender), risk factors (e.g., sexual activity, number of sexual partners, condom use, drug use and HIV test history), knowledge and attitudes towards self-testing, concerns, willingness to pay, challenges, preferences for counselling and treatment linkages, and perceived barriers to self-testing. All data were entered in Microsoft ACCESS and analyzed in STATA version 11 (STATA Corp., TX, USA).

The primary end point was the agreement between the self-test performed by the study participant and the test performed by the HCP on the student. Secondary end points on acceptability and preference were analyzed using proportions. Additionally, open-ended qualitative questions on concerns, challenges, and barriers in the QES allowed students to freely voice their opinions and concerns regarding oral self-tests and served to complement quantitative findings [23, 24]. A thematic analysis was performed to gain further insight on student responses (Table 2). Categories were developed to reflect emerging themes from recurring keywords and similarities in concepts [25]. Each response was assigned to one or more category (e.g., test accuracy) depending on the number of concepts expressed within the response. To reduce misclassification, a second researcher read through the responses and discrepancies on assignment of categories were resolved through discussion.

## 3. Results

Of 232 students approached, 33% (*n* = 76) did not consent, and a further 5% (*n* = 11) did not complete all study procedures, thus giving a final sample size of 145, all of which had been confirmed to be seronegative before participation. Demographic and risk characteristics of participants are summarized in Table 1. Participants were young (median age: 22 years), predominantly female (61%, *n* = 88), unmarried (97%, *n* = 141), with 47% (*n* = 68) being out-of-province or international students. A third of participants (33%, *n* = 48) had never tested for HIV in the past, but a majority (87%, *n* = 125) were sexually active. In the last six months, half (50%, *n* = 71) reported multiple sexual partners and a quarter (25%, *n* = 36) reported having had unprotected casual sex. Casual sex was defined as sex with a person who was not a regular or established partner (less than three months).

With respect to feasibility of self-test conduct, results from the self-tests performed by students had 100% agreement with the test performed at the point-of-care by the HCP. No false negative or indeterminate results were recorded with either test. Regarding acceptability of self-testing, 81% (*n* = 117) of students preferred the oral self-test to the laboratory test, and 87% (*n* = 126) were confident about interpreting their test results themselves. Almost all (99%, *n* = 143) found the test to be convenient. For posttest counselling and linkages, community clinics (78%) were the top choice, followed by phone (54%), pharmacies (33%), and the Internet (30%). Approximately 41% (*n* = 60) were comfortable with either anonymous or face-to-face counselling, whereas 39% (*n* = 56) preferred face-to-face and 16% (*n* = 23) preferred anonymous counselling. Approximately 74% (*n* = 107) expressed willingness to buy self-tests OTC, but only 28% (*n* = 40) were willing to pay more than $20.

Qualitative findings were tertiary to our study, and thematic analyses of the two open-ended questions on student opinions and concerns are summarized in Tables 2 and 3. The main themes that were retained reflected students' concerns regarding the following issues: (1) self-test accuracy, (2) acceptability, (3) linking seropositive subjects with counselling and medical care, and (4) increase in HIV testing with the self-testing option (Table 2). Concerns regarding accuracy were repeatedly raised, although some predicted that, once the self-test had proven accuracy, it would be preferred over the conventional method. The “how” and “where” of linking seropositive self-testers to medical and psychological care concerned respondents. Students seemed confident that the self-testing method was perceived to be easier and more accessible than conventional testing allowing access and expansion of HIV testing.

The second open-ended question solicited opinions on the general concept of self-testing and its widespread implementation, and the students' responses were focussed on the following issues: (1) test administration, (2) time-saving process, (3) accessibility, and (4) privacy (Table 3). The idea of using oral fluid was highly appealing, as was the ability to save time—as presentation to a health facility is not required for self-testing—and self-test results were available within minutes versus weeks. Making the test accessible to the public was considered important with regards to increasing the number of people and frequency for HIV testing. Many participants thought pharmacies could be a suitable venue to make the self-test available. Finally, respondents valued the privacy afforded by the oral self-tests.

## 4. Discussion 

This cross-sectional study generated evidence on feasibility of operationalization of an unsupervised self-testing strategy in students. Feasibility, defined as a high agreement between the self-test performed by the students and the test repeated by the HCP, reflects on the ability of participants to perform and interpret self-test results without errors. This successful conduct of self-tests may have been related to the use of instructional videos and pamphlets provided to literate participants (students) who could comprehend them, as well as the fact that, by accepting to participate in the study, these participants showed interest in conducting self-tests by themselves [22].

Our secondary findings revealed that students were willing to purchase oral self-tests OTC, which they found preferable to facility based tests, although they expressed concerns about the accuracy of self-test and the linkage to care process. A high preference for oral samples suggests the desire for noninvasive and convenient oral self-tests instead of finger stick tests. In terms of preferences for counselling, 41% (*n* = 60) of participants were comfortable with either anonymous or face-to-face counselling, while 39% (*n* = 56) and 16% (*n* = 23) preferred face-to-face and anonymous counseling, respectively. This diversity in opinion as to the desired approach to counselling was also observed in the preferred setting for counselling, although community clinics were the option with which most participants were comfortable. This pattern suggests the need to tailor linkages to counselling based on needs and an offer of personalized counselling based on preferences of students.

In terms of cost preferences, students expressed willingness to buy HIV self-tests OTC, with a preferred price point of <$20. Cost concerns are key to the uptake of self-tests worldwide and may vary according to context. As an example, the current US sale price of $40 exceeds what most of our study population would have been willing to pay for the self-test, which might impede its uptake, if offered in future to this population.

Some study participants also expressed a concern regarding the diagnostic accuracy of oral tests. This concern was explored in a recent meta-analysis published by our group in 2012 [26]. This meta-analysis compared the diagnostic accuracy of oral tests versus blood tests and demonstrated a 2% lower sensitivity of oral tests compared to blood tests [26].

In this respect, highlighting the limitations of this oral antibody test in picking up new infections within 90 days would be very important. This is especially true for those students that may test themselves immediately after a recent exposure and find themselves falsely negative. The current version of the self-test sold OTC in the US emphasizes the need to test only at or after 90 days of possible exposure. Although most at-risk populations that are frequent testers may be aware of this fact, it may not be the case for new testers and those at lower risk like our student populations.

Students represent a subset of “worried well” populations who may seek self-tests if they are available OTC at pharmacies [27]. This could be due to the following factors: (a) health systems issues (e.g., difficulty in accessing health services due to long waiting time), (b) lifestyle preferences, (c) convenience of oral self-tests, (d) time savings with rapid tests, and lastly (e) confidentiality and privacy offered by self-tests.

Our study findings are consistent with the evidence from studies that evaluated self-tests in different populations worldwide (i.e., Spain, USA, and Malawi) [15, 17, 18]. A high agreement of the self-test result with the test repeated by the HCP and a high preference for face-to-face counselling were also noted in other studies [15, 18, 20, 28], as were convenience, speed, privacy, and anonymity of self-tests as facilitators [16, 18, 20, 21, 28, 29].

## 5. Implications

Our study results may be of interest for other populations with a low level of perceived HIV risk, but the study needs to be repeated in populations at high risk of acquiring infection.

This pilot study which used a mixed methods approach was informative in evaluating the feasibility of conducting an unsupervised self-testing strategy using a rapid oral HIV test in Canada. More evidence from large sized implementation research studies or controlled trials needs to be collected in the near future so as to inform public health agencies in Canada on the use of self-testing. Furthermore, the information gained from the pilot study is relevant for planning tailored self-testing initiatives for at-risk populations. These at-risk populations (e.g., immigrants from endemic settings, aboriginal populations from the rural North, and men who have sex with men) have diverse socioeconomic status, preferences, and lifestyles. These parameters need to be accounted for in planning self-testing initiatives and when evaluating if the supervised or unsupervised approach is the best strategy for a specific population. Whichever strategy is chosen, availability of expedited counselling and linkage systems will be key to mobilizing marginalized populations with limited access to health care professionals to consider self-testing. As an example, populations in the rural North, which report a high incidence of HIV, experience extreme weather conditions that hamper timely access to testing and face stigma associated with HIV testing in tightly knit communities. Therefore, setting up culturally sensitive self-testing strategies would be key to improving the uptake and may potentially lead to an earlier diagnosis and improved management of HIV infected individuals and potentially decrease transmission.

This pilot study was intended to demonstrate feasibility of a self-testing strategy, operationalized in a population that could in all likelihood purchase OTC HIV self-tests were they made available. However, our study is the only one of its type to have been conducted in Canada, and while community-based studies such as ours may be informative and useful in addressing how to bring these populations to test, they can only be repeated in a real world setting if self-tests are approved in Canada. Operationalizing timely linkages to counselling and treatment referrals from remote settings will be more relevant with the approval of such tests [30–32].

## 6. Limitations

Interpretations of our study findings are subject to limitations. Our study enrolment period corresponded with a staff and student strike at the study university, which impaired our recruitment efforts. Study procedures called for two trips to the clinic, which yielded loss to followup of participants. A convenience sample of students was enrolled, raising the potential for possible selection (volunteer) bias. Finally, research with investigational devices such as the test we used limits the documentation of the full spectrum of concerns and challenges that may arise with an approved self-test.

## 7. Conclusion 

To conclude, an unsupervised oral self-testing strategy for HIV was feasible to operationalize in a healthcare setting in Canadian students. Although these findings support the use of self-testing, this pilot study calls for further exploration of offering self-testing strategies in real life settings in larger samples of high-risk populations in Canada.

## Figures and Tables

**Table 1:** Demographic characteristics and STI risk level of study population (*N* = 145).

Category	Percentage % (*N*)
Gender	
Male	38.9% (56)
Female	61.1% (88)
Education	
CEGEP/college	20.6% (30)
Undergraduate degree	45.5% (66)
Graduate degree	20.0% (29)
Medical degree	0.7% (1)
Vocational or Trade School	13.1% (19)
Residence	
Montreal	49.7% (72)
Quebec but not Montreal	3.4% (5)
Other Canadian province	18.6% (27)
United States of America	13.8% (20)
Other country	14.5% (21)
Marital status	
Married	2.7% (4)
Notmarried	97.2% (141)
No. of sexual partners in the last six months	
None	12.6% (18)
1	37.8% (54)
2–5	39.2% (56)
More than 5	10.5% (15)
Sexual behavior	
Never had a casual partner	20.0% (29)
Protected sex with casual partner at some point in life	66.2% (96)
Unprotected sex with a casual partner at some point in life	47.6% (69)
Protected sex with a casual partner in the last 6 months	46.2% (67)
Unprotected sex with a casual partner in the last 6 months	24.8% (36)
Unprotected sex with a casual partner in the last 6 months under the influence of alcohol	18.6% (27)
Unprotected sex with a casual partner in the last 6 months under the influence of drugs	7.6% (11)
Condom use	
Every single time	27.0% (39)
Almost every time	25.7% (37)
Sometimes	11.1% (16)
Rarely	6.9% (10)
Never	17.4% (25)
No sexual partner in the last six months	11.8% (17)
Persons reporting injection drug use	
Yes	0.7% (1)
No	97.9% (141)
Do not know	1.4% (2)
Sex toys use	
Yes	7.6% (11)
No	91.7% (132)
Other	0.7% (1)
Blood transfusion	
Yes	2.1% (3)
No	97.9% (141)
STI history	
Never had an STI	80.7% (117)
Currently have an STI	2.8% (4)
STI in the last six months	4.8% (7)
STI in past but not in the last six months	8.3% (12)
Other	2.1% (3)
HIV testing history	
Less than six months ago	21.5% (31)
Six months to one year ago	17.4% (25)
One to two years ago	18.0% (26)
More than two years ago	9.0% (13)
Never	33.3% (48)
Reason for not having tested for HIV in past	
Previously tested, does not apply	63.5% (92)
Do not think of being at risk	28.9% (42)
Do not want to have it on medical records	0.7% (1)
Other	9.0% (13)

**Table 2:** Select issues and concerns on oral self-testing versus conventional blood testing* (*N* = 145).

Test accuracy	Acceptability	Concerns with linkages	Increase of HIV testing
“I prefer conventional right now because I am sure the result is more accurate than the self-test.” “Only 99% effectiveness of the test might make me still want a blood test.” “I am more confident the results of the conventional blood test and plus they might be able to test for other things at the same time.” “I presently would not purchase self-test since they have not been used for long time. So I would doubt the accuracy. But if after 1 or 2 years, studies showed that it is accurate, I would prefer the self-test.” “Concern over user error with oral self-test.”	“For people who hate needles it is an excellent alternative; quick, non-invasive, private and easy-to-do.” “The oral test is easier, less painful and less time consuming...” “Easy and straight forward.” “I like the fact that the oral test is easy and low stress. Taking a test outside of an institutional setting is less nerve wrecking.”	“But the concern is lack of counselling in event of positive result when self-testing.” “If the self-test is positive, what does one do? There should be steps listed of what to do afterwards because a positive result would be very disconcerting.” “Also it seems that receiving the results from a doctor is safer, as receiving positive result should not happen alone.” “Could lead to more people finding out their HIV status who might not otherwise get tested. But very important that appropriate counseling/education resources in place to support individuals who find out their positive status through a self-test.”	“I think it is a great idea, I would have gotten tested sooner it I knew it were an option.” “This will make HIV testing much more accessible to people. I think that the hospital/clinic setting for blood tests discourage many people.” “This sounds amazing, especially for regions where getting HIV testing is more difficult. I hope this becomes cheaply available in developing countries.”

*Participants were asked an open-ended question: “If you have other comments regarding oral self-testing versus conventional blood testing, please let us know.”

**Table 3:** Select reasons expressed by participants for why self-testing should be made available* (*N* = 145).

Ease of administration	Process time and convenience	Accessibility	Privacy
“It is easy to self administer and can be accessed simply.” “Quick and simple. Seems accurate. If positive gets quicker treatment.” “It is fast and pain free.” “There are people who are scared of needles, faint at the sign of blood; its easy and quick, provides privacy.” “No waiting for an appointment and getting poked with needles.”	“It is much more convenient than the blood test and it is faster. No need to make an appointment or to go to a clinic as a first step.” “You get results right away and you can do the test frequently without having to see a doctor which usually means a long waiting time.” “I can show results more quickly in case someone really needs to get tested, and some people may prefer this as a preliminary method.” “It is much more convenient and eliminates anxiety involved in waiting for blood results for 2 weeks.”	“It is easier if more accessible in pharmacies for people to test themselves if they have any concerns.” “If self-testing is sold in pharmacy, I think more people would be willing to do it instead of going directly to a clinic.” “I also think it would lead to more frequent testing per individual.” “More people will have access to the test, those who do not have family doctors or don't know of STI testing sites.” “It is useful for people without easy access to a clinic.”	“It gives discretion and privacy to people when they need a fast response system compared to the conventional blood test.” “It allows people to get the news privately.” “It gives people more privacy. A person who is ashamed to go to general hospital will find their HIV status at an early time, which can save his life.” “It enables more privacy when testing for something very personal.”

*Participants were asked an open-ended question: “Given your experience in this study, do you think self-testing for HIV is a good idea? Do you think it should be an option made available to people?”
